# Performance evaluation of a new type of commercial silicon detectors for photon beam dosimetry

**DOI:** 10.1002/acm2.70719

**Published:** 2026-07-31

**Authors:** Indra J. Das, Meisong Ding, George X. Ding

**Affiliations:** ^1^ Department of Radiation Oncology Northwest Memorial Hospital Northwestern University Feinberg School of Medicine Chicago Illinois USA; ^2^ 1513 Anna Marie Cir Ambler Pennsylvania USA; ^3^ Present address: Department of Radiation Oncology Vanderbilt University School of Medicine Nashville Tennessee USA

**Keywords:** characterization, photon beam, silicon diode detector, small field detector

## Abstract

**Background:**

Modern radiotherapy relies on advanced treatment techniques that utilize a large number of small fields, for which maintaining dosimetric accuracy is often challenging. Most vendors have introduced microdetectors suitable for small field dosimetry. Sun Nuclear has developed two silicon diodes with a sensitive volume of 0.35 mm^3^ that are intended to replace their current EDGE diode detector. Detector characterization is required before it can be used for clinical practices.

**Purpose:**

Two Sun Nuclear silicon detectors (unshielded model 1048 and shielded model 1049) are evaluated to characterize their relative dosimetric performance in megavoltage photon beams, with particular emphasis on small fields.

**Methods:**

Sun Nuclear silicon detectors are identical in physical dimensions except that model 1049 is shielded. These detectors are investigated in Varian TrueBeam photon beams (6 MV, 6 MV FFF, 10 MV, 10 MV FFF and 15 MV) for dose linearity, dose rate linearity, thermal stability, and usability for small fields across multiple photon energies. The measured data are benchmarked against Monte Carlo (MC) simulation using EGSnrc for depth dose, profile and output factors. Additionally small field correction parameters $k_{Q_{clin},Q_{msr}}^{f_{clin},f_{msr}}$ were determined experimentally and compared to Monte Carlo data.

**Results:**

These detectors exhibited excellent dose linearity across five photon energies, with only a minor deviation of approximately 1.5% at doses below 2 MU. The dose rate linearity remained stable from 100 MU/Min to highest dose rate for all beam energies with a slight upward trend of 1.5% for lower dose rates. Both detectors are also temperature independent, within 1.0% over the range of 4°C–40°C. Depth dose and profiles measurements showed good agreement with Monte Carlo data across the applicable field size range. For small field dosimetry, shielded detector exhibited a pronounced signal reduction for fields smaller than 2 cm, resulting in large correction factors, whereas the unshielded detector demonstrated favorable and stable performance.

**Conclusions:**

Both silicon diodes have excellent dosimetric characteristics, providing reproducible data with signal linearity. They also provide a large signal (nC). For smallfield photon dosimetry, the unshielded detector yielded superior performance across all investigated energies.

## 1 INTRODUCTION

Solidstate detectors, primarily silicon diodes, have evolved for use in radiation beams because of their favorable dosimetric characteristics, including an efficiency nearly 18,000 times greater than that of ionization chambers.^1^, ^2^ This increased efficiency arises from the higher density of silicon and its lower ionization energy compared with air. Rickner et al. provided detailed descriptions of the use of silicon diodes in radiation dosimetry,^3^, ^4^ which were later extended by the AAPM Task Group on in vivo dosimetry.^5^ A substantial body of literature describes the design, characteristics, and clinical use of diode detectors in radiation therapy.

Silicon diodes have become an attractive choice for detectors especially in smallfield dosimetry, due to their small sensitive volume and high signal per unit dose. Virtually every radiation therapy vendor now offers diode detectors due to their small sensitive volume, high signal output, mechanical robustness, waterproof design, and capability for realtime dosimetry. However, their relatively high density and effective atomic number necessitate the application of significant correction factors.^6^, ^7^ To address these limitations, silicon detectors have been further miniaturized into microSilicon detectors, which exhibit favorable characteristics in small fields and electron beams.^8^, ^9^, ^10^ The use of microSilicon detectors, with reduced correction requirements, has been widely documented for conventional linear accelerators and MRlinac–based applications.^11^, ^12^, ^13^, ^14^, ^15^, ^16^

**FIGURE 1:**
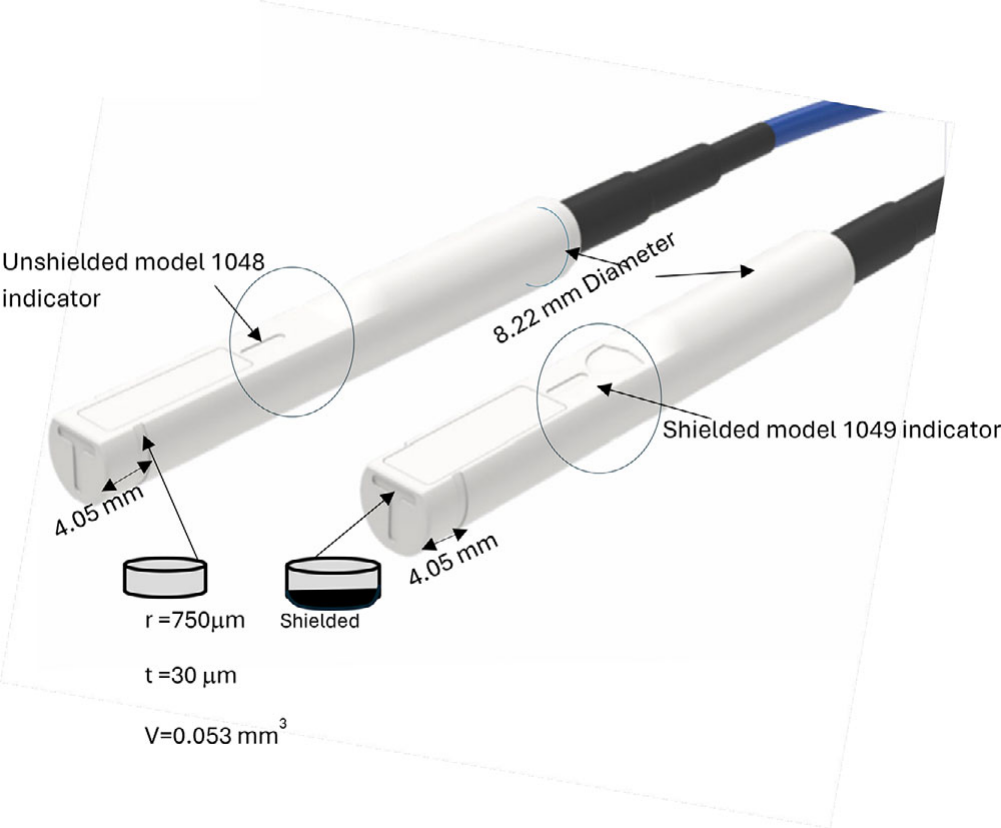
Schematic of the Sun Nuclear Silicon detectors (SunSILICON), both have identical design and dimension except Si active disk in model 1049 is shielded which is shown in the figure. Various components of the housing assembly are shown such as etching (in circle) to differentiate the model. Figures are not up to scale.

Sun Nuclear (Melbourne, FL) has pioneered the clinical use of diode technology across multiple product lines, including daily quality assurance systems, patient specific QA system such as MapCHECK and ArcCHECK. The Edge detector (Sun Nuclear), introduced in 2005, employs a single diode with a sensitive area of 0.8 × 0.8 mm^2^ and has become a widely used detector for beam scanning, relative dosimetry, electron beam measurements, smallfield radiosurgery, and MRlinac applications. The Edge detector is now being replaced by the SunSILICON diode series, which is commercially available in two models (1048 and 1049). Model 1048 is unshielded and intended primarily for electron beam and smallfield photon dosimetry, whereas the SunSILICONP model 1049 is shielded for generalpurpose photon beam dosimetry. Both detectors share an identical geometric design, as shown in Figure 1, and are constructed from high‐equivalency (HE) solid water model 557 proprietary of Sun Nuclear.

During fabrication, these detectors are annealed and preirradiated to kGy dose levels using a Co60 beam to stabilize their response. Anders et al. evaluated the sensitivity of these detectors following cumulative radiation doses of 16, 32, and 48 kGy and reported no measurable loss in signal, indicating minimal radiation induced damage.^17^

It is not clear why Sun Nuclear is replacing Edge detector with two different new detectors for photon and electron beam dosimetry. Characterization of any new detector design especially for dosimetry and field reproducibility, sources of error, are essential prior to clinical implementation. While Anders et al. provided an initial detector characterization from the manufacturer's perspective,^17^, ^18^ the present study extends this work by providing an independent and comprehensive evaluation of SunSILICON detector performance in megavoltage photon beams, with particular emphasis on smallfield dosimetry across multiple photon energies.

## 2 MATERIALS AND METHOD

### 2.1 Detector description

The SunSILICON detectors, models 1048 and 1049, have identical external designs in which the sensitive volume is a p‐type silicon diode placed within a rather irregular solid‐water geometry compared to the brass housing used in the EDGE detector. The outer dimension of the housing is an 8.22 mm diameter that is tapered towards the end (Figure 1). SunSILICON model 1048 is unshielded and designed primarily for electron beam and small field photon dosimetry. SunSILICON P model 1049 is shielded with a thin layer of high atomic‐number (Z) alloy (tungsten, tantalum and gold; composition proprietary) to remove low energy scatter but still maintain water equivalency. The shielding is used primarily to improve detector response in medium to large fields and reduce overresponse in large fields. This model is intended to replace the Edge detector for general‐purpose photon beam dosimetry.

The sensitive volume of the detectors is a silicon (Si) disk with a radius of 750 µm and a thickness of 30 µm corresponding to a volume of 0.053 mm^3^. The entire device is embedded in high‐equivalency (HE) solid water model 557 proprietary to Sun Nuclear. Figure 1 shows the housing of the detectors: the top image is the unshielded detector and the bottom image shows the shielded detector. The position of the center of the Si‐disk is located 4.05 mm from the detector tip at a depth of 0.125 g/cm^2^ which can be treated as the entrance window. Since both detectors have identical shape and size, a visual distinction can be made with the etching on the detectors as shown by the circle region (Figure 1). The detectors come with either BNC or TNC connectors depending on user preferences.

The sensitive volume is located approximately at a depth of 1.25 mm and 4.05 mm from the front edge. For ease of setting up in a water phantom, the detectors are supplied with cap that includes crosshair corresponding to the same location indicated by the arrows. Anders et al.^18^ provided effective point of measurement (EPROM) to be 1.2 ± 0.3 mm and 1.45 ± 0.3 mm for unshielded and shielded SunSILICON detectors, respectively which were used in the measurements in this study.

### 2.2 Measurements

Anders et al.^17^, ^18^ have provided some characteristics of these two detectors. To avoid redundancy, the present study focused on detector properties that were either insufficiently characterized or required further validation, particularly for smallfield photon dosimetry. All measurements were performed on a Varian TrueBeam linear accelerator with five photon energies (6 MV, 6 MV FFF, 10 MV, 10 MV FFF and 15 MV). Both detectors were mounted in a water tank for simultaneous exposure and data collection. A high‐fidelity SuperMax electrometer (Standard Imaging, WI) having dual channel for simultaneously charged collections was used. The bias on electrometer was set to zero that provided negative signal. For time saving and repeatability the triggered data collection option was used. The electrometer could measure charge in the range of fC and has auto gain that was used in conjunction with SunSILICON detectors.

For dose linearity and dose rate dependence, all measurements were performed in reference condition (10 × 10 cm^2^, *d*_max_ at 100 cm SAD) for all energies. For temperature dependence, a small plastic water bucket (30 × 30 × 20 cm^3^) was used. The bucket was placed on a 6 cm solid water phantom on the treatment table. The gantry was rotated to 180° to maintain the same amount of attenuating materials in the beam as the water content varied depending on the addition and removal of hot and cold water. Temperature was monitored using a calibrated digital thermometer with an accuracy of 0.1°C. Water temperature was adjusted by adding ice cubes and later hot water. The temperature was varied in the range of 4°C–45°C. Two sets of temperatures were recorded, one before and the other after exposure. An average of these two temperature readings was used for analysis. Readings were collected simultaneously for 100 MU by changing the water temperature. Readings were collected for 100 MU for each temperature setting.

Depth dose and profiles were measured with IBA Dosimetry 3D water scanning systems (commonly referred to as the “Blue Phantom”) with software version 9.2.49.0. The measurement accuracy of its motion is 0.1 mm. Data were collected with these two detectors in the context of small fields as data for large fields have been provided by Anders et al.^17^, ^18^ Additionally, data from a PTW microSilicon detector were collected for comparison, as it has demonstrated to have high spatial resolution and favorable performance in small fields.^8^

An ideal detector should provide dosimetric parameters, depth dose, tissue maximum ratio, off‐axis ratio, and output factor with minimum perturbation. It is well known that in nonequilibrium conditions such as small fields, ratio of radings cannot be assumed to represent the ratio of dose. Therefore a correction factor was introduced by Alfonso et al.^19^ and described in detail in various reports.^7^, ^20^

(1) $$\frac{D_{1}}{D_{2}}\neq \frac{M_{1}}{M_{2}}$$

(2) $$\frac{D_{1}}{D_{2}}=\frac{M_{1}}{M_{2}}\;•\left[k_{Q_{clin},Q_{msr}}^{f_{clin},f_{msr}}\right]$$

where *D* is radiation dose, *M* is measured reading (detector response). The correction factor ($k_{Q_{\mathrm{clin}},Q_{\mathrm{msr}}}^{f_{\mathrm{clin}},f_{\mathrm{msr}}}$) represents detector specific values for *f_clin_*, clinical field size, *f_msr_*, machine specific field size and *Q* is the beam quality. TRS‐483 provided special name for FOF as defined in Equation (3).

(3) $$\mathrm{FOF}=\left[\Omega_{\;Q_{clin},Q_{msr}}^{\;f_{clin},f_{msr}}\right]=\frac{M_{1}}{M_{2}}\;•\left[k_{Q_{clin},Q_{msr}}^{f_{clin},f_{msr}}\right]$$

FOF was measured for these two detectors in photon beams.

For small field, Field output factor (FOF) in small field was measured using IBA scanning water phantom sequentially with each detector. Measurements were made from 5 to 100 mm square fields made with collimating jaws. Detectors were kept at 100 cm SAD for all measurements. The reproducibility of field size for TrueBeam even in small fields is very high (±0.01 mm).^21^

### 2.3 Monte Carlo simulation

Monte Carlo simulations were performed using the EGSnrc^22^, ^23^ Monte Carlo system with user code BEAMnrc/DOSXYZnrc^24^ for generating the realistic incident beams and dose calculation. The simulated beams from Varian TrueBeam were based on the phase space files provided by Varian (Varian 2014, version 2.0) that was experimentally validated.^25^ The simulated incident beams were stored in phase‐space files and used as inputs for dose calculations. The parameter settings used in the Monte Carlo simulations were AE = ECUT = 0.521 MeV, AP = PCUT = 0.010 MeV. The calculation grid size was 0.025 cm × 0.025 cm = 0.000626 cm^2^. The accuracy of the Monte Carlo simulated beams was validated by measurements as published in several recent publications.^15^, ^26^

The FOF data were computed by Monte Carlo simulation as presented in previous publication.^27^ Since $k_{Q_{\mathrm{clin}},Q_{\mathrm{msr}}}^{f_{\mathrm{clin}},f_{\mathrm{msr}}}$ is not available for these detectors, they were derived from the Monte Carlo data for field size and beam energies.

## 3 RESULTS

### 3.1 Dose linearity

**FIGURE 2:**
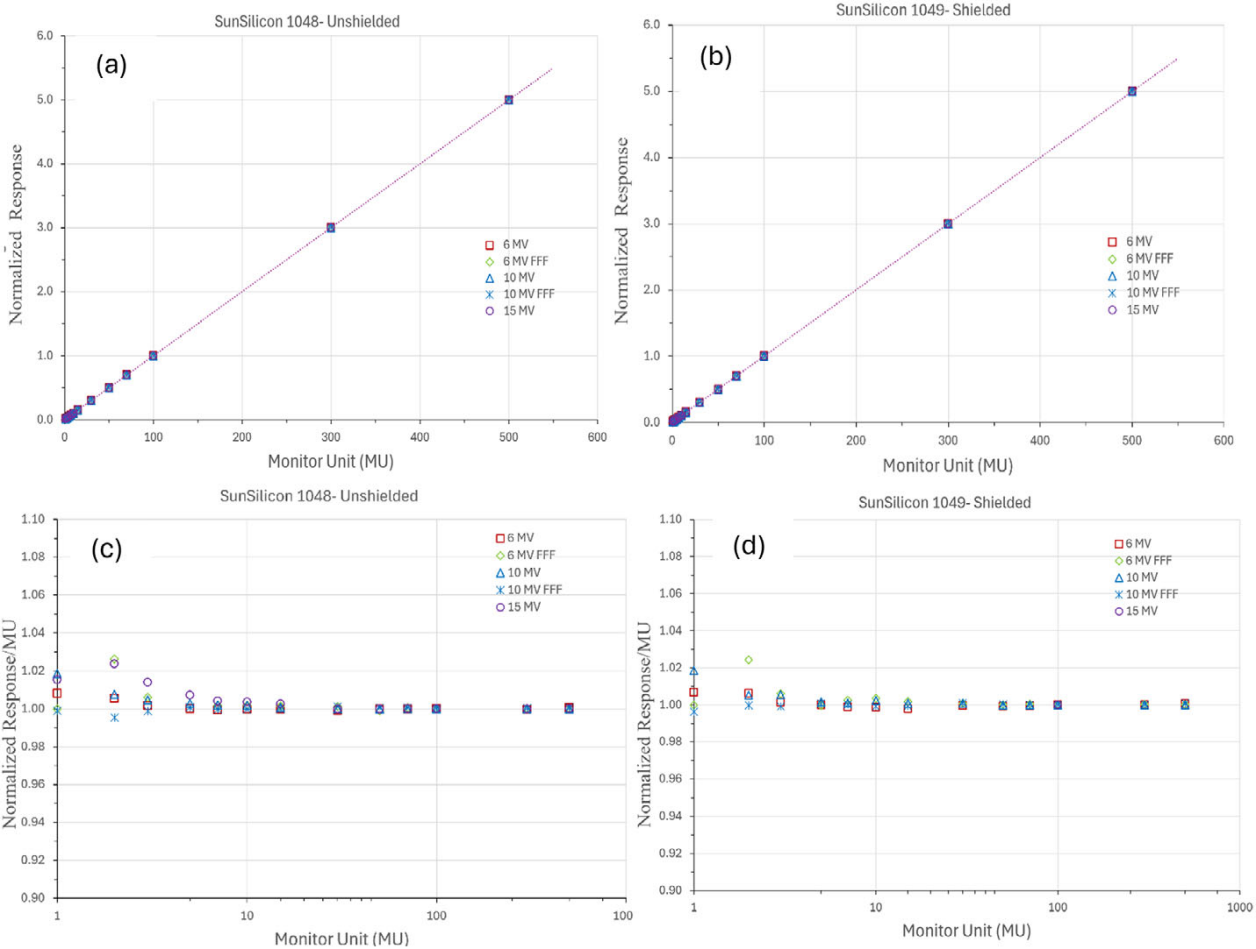
Signal linearity versus MU for unshielded and shielded detector; (a) unshielded (b) shielded detector for all 5 beam energies. Normalized response/MU plotted on logarithmic scale for all energies (c) unshielded and (d) shielded detector.

A radiation detector should provide linear signal with respect to dose and beam energies, which was tested for both SunSILICON detectors. Figure 2(a,b) shows the normalized signal response for five photon energies including 6 MV FFF and 10 MV FFF which are predominantly used in IMRT and VMAT treatments. Data are extremely reproducible as all five symbols cannot be distinguished on the plot. The reproducibility of signal with repeated measurement was very high, with variation < 0.2%except for very low dose (10 MU) where variation was <0.01%. Since the data were reproducible, statistical error bars were not discernible and are therefore not shown in Figure 2.

When the normalized signal/MU is plotted on a logarithmic scale, small changes are visible for <3 MU for both detectors and across all energies as shown in Figure 2(c,d). Error bars cannot be plotted due to identical signal reading. The signal variation is approximately 2% for <3 MU and 0.5% for >3MU for both detectors. No statistically significant differences are observed across beam energies. This observation was also reported by Anders et al.^17^ However, they observed energy‐dependent differences, where 15 MV shows a downward trend with a dose variation of nearly 0.3% at 50 Gy, which was not observed in 6 MV noticed differences in energies where 15 MV shows downward trend with dose of nearly 0.3% at 50 Gy but this was not observed in 6 MV. Such remarkable reproducibility is due to the exceptional fidelity of the TrueBeam system and the high degree of quality assurance and manufacturing precision applied to the detectors. By comparison, older accelerators have shown dosimetric variations as large as 25% at very low MU settings.^28^

### 3.2 Dose‐rate linearity

**FIGURE 3:**
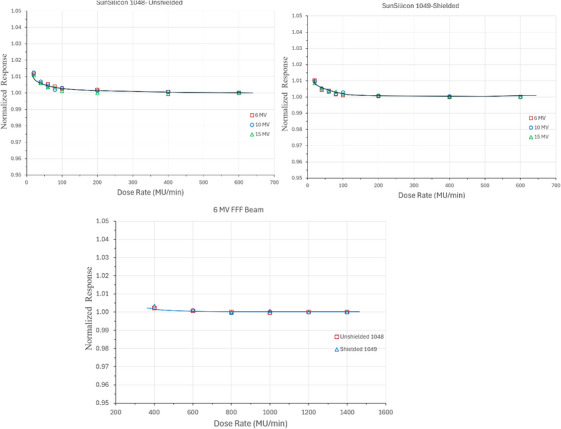
Dose rate linearity plotted as normalized response vs. dose rate; (a) for unshielded model 1048 and (b) for shielded model 1049 for standard beam energies and (c) shows data for FFF beam for both detectors.

Doserate dependence is a critical parameter because modern treatments employ a wide range of dose rates. Figure 3 shows the normalized detector response as a function of dose rate for both SunSILICON detectors. For standard flattened beams, the response was stable over the full range of available dose rates. For FFF beams, a slight upward trend (∼1.5%) was observed at lower dose rates. Overall, both detectors demonstrated excellent doserate linearity, indicating that no doserate correction is required for routine clinical use.

### 3.3 Angular dependence

Angular dependence was not directly investigated in this study due to the noncylindrical geometry of the SunSILICON detectors, which limits the practical relevance of such measurements. Previous work has shown angular variations of within ±1% for rotation angles up to ±28°.^18^ In routine clinical practice, these detectors are used in water phantoms with the beam incident perpendicular to the detector axis, and angular dependence is therefore expected to have negligible dosimetric impact.

### 3.4 Temperature dependence

**FIGURE 4:**
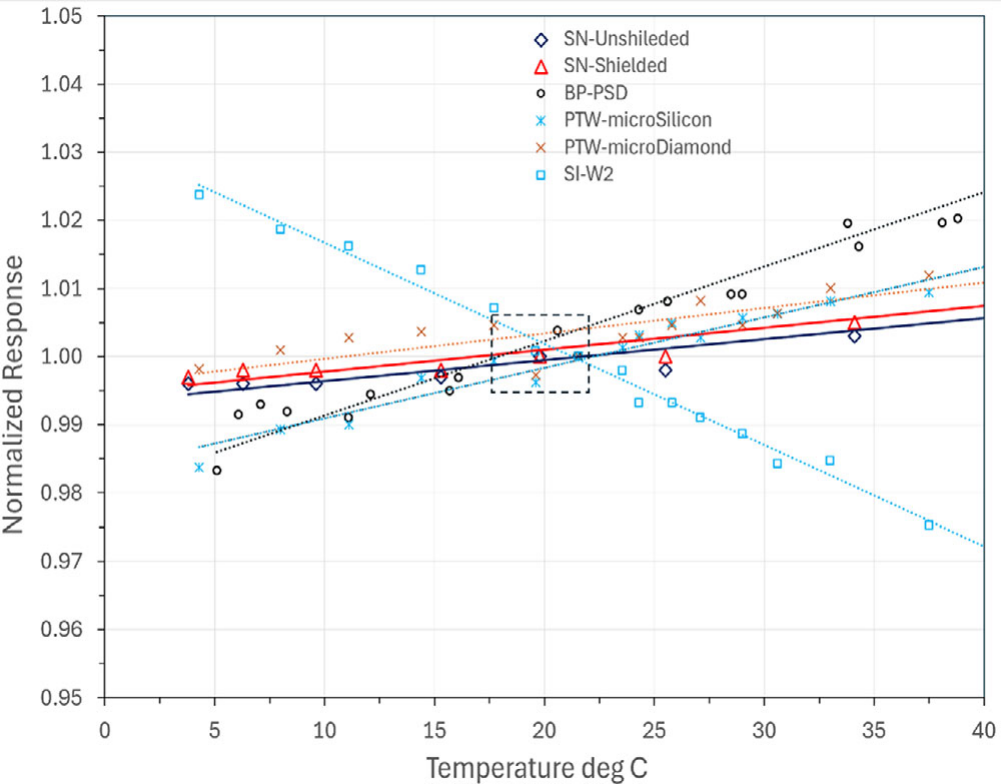
Temperature dependence of both SunSILICON detectors (solid lines) along with other solid state detctors. Data are reproduced from various publications.^8^, ^29^, ^30^, ^31^

The temperature dependence of both SunSILICON detectors is shown in Figure 4, along with comparative data from other solidstate detectors reported in the literature. To compare the SunSILICON response with other detectors, data from published literatures for PTW‐microsilicon,^8^ BluePhysics PSD (BP‐PSD),^29^ PTW‐microDiamond,^30^ and Standard Imaging W2^31^ are shown. Most solidstate detectors exhibit a positive temperature coefficient, whereas plastic scintillation detectors show a negative trend, likely due to temperature dependent refractive index effects. For SunSILICON diodes variation is 0.2% and trend from 4 to 40 deg in 0.4%. Within clinical conditions (18°C‐22°C) these detectors can be treated as temperature independent within ±0.5% as shown in the box (Figure 4) however, SunSILICON values are very small (0.01%).

### 3.5 Depth dose

Percent depth dose (PDD) variation with detectors and field size is well known.^32^ This is due to detector sensitivity to low energy scatter in large fields. SunSILICON shielded detector is meant to provide invariant response in field size. This has been shown to be nearly true where these detectors were compared with Edge detector and microdiamond. For shielded detector (model 1049) there was ±1% difference compared to Edge detector.^18^ For unshielded (model 1048) PDD differences peak at a depth of 15 cm and for large fields (40 × 40 cm^2^) with a magnitude of 4.5%. For reference field size most of these detector provided identical results.^18^

**FIGURE 5:**
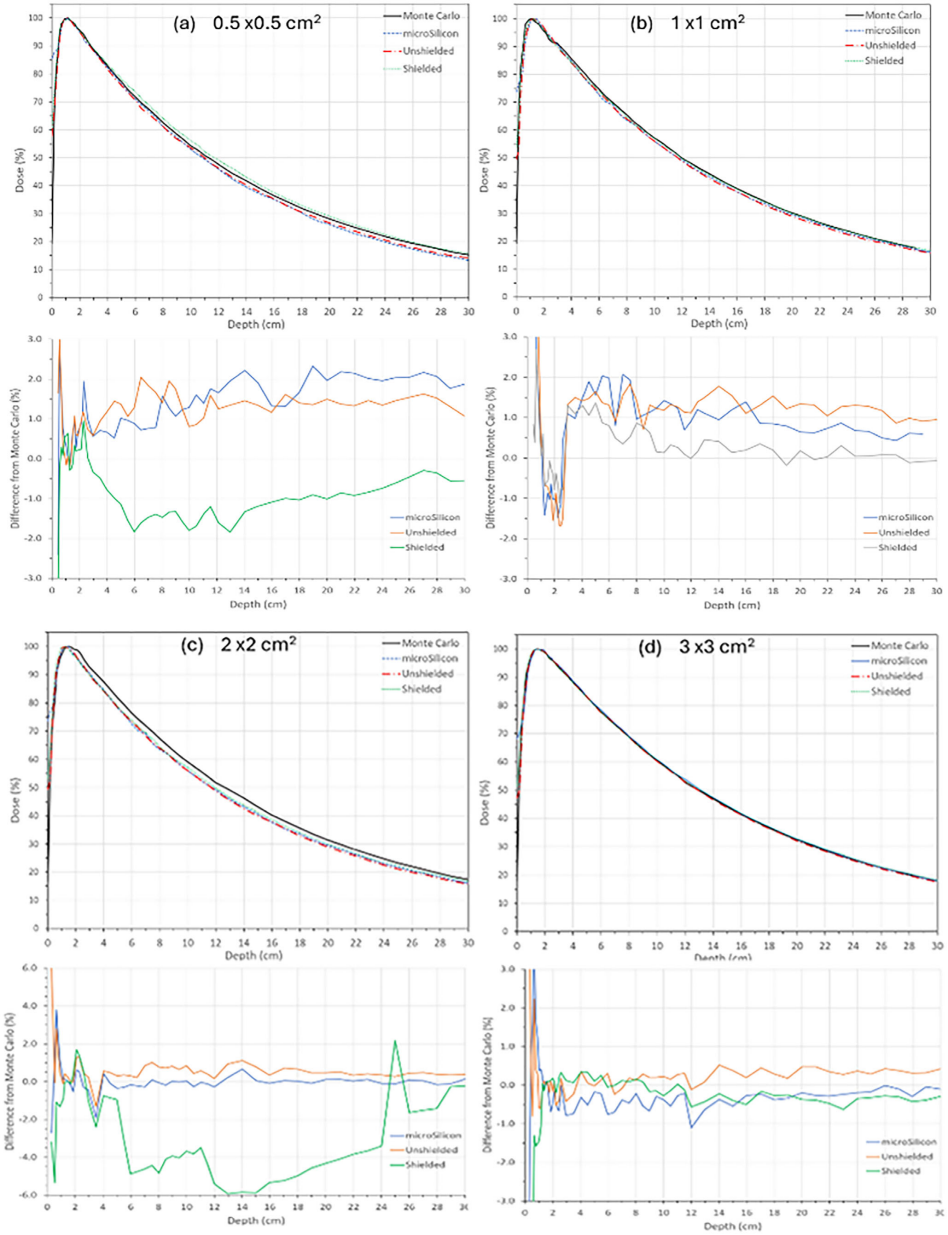
Depth dose for 6 MV beam for 0.5 (a), 1.0 (b), 2.0 (c) and 3.0 cm (d) square field. Difference plot is shown below each panel compared to MC data.

In this study depth dose is taken for small fields ≤3 × 3 cm^2^ which are shown in Figure 5 compared to the MC data. For comparison these data were also taken with microSilicon at the same time on same machine which is shown in Figure 5. The differences are minimal in pictorial form; however, difference plots provide the magnitude of the difference as shown below for three detectors. The differences could be due to setup uncertainty, centering, step size, and EPOM. It is known that voxel size used in Monte Carlo has very little effect for PDD but Monte Carlo history has effect on the smoothness of the curve.

### 3.6 Beam profile

**FIGURE 6:**
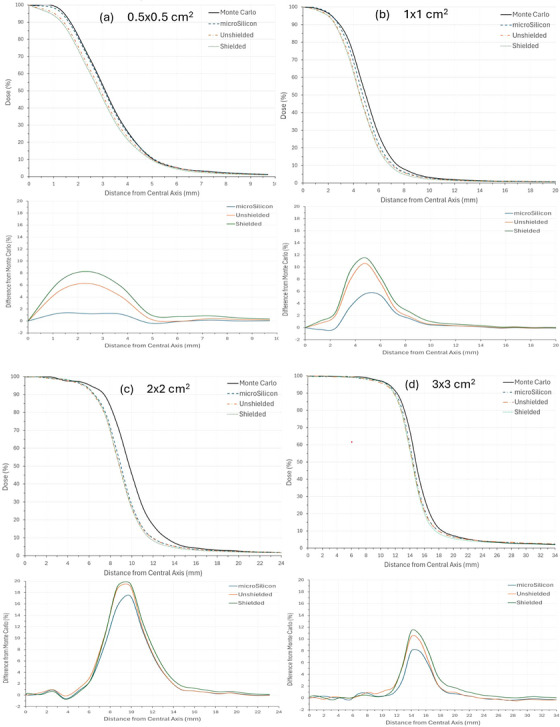
Beam profiles for 6 MV beam for 0.5 (a), 1.0 (b), 2.0 (c) and 3.0 cm (d) square field for three detectors. Difference plot is shown below compared to MC data.

Beam profile is a critical parameter for modern treatment, especially in stereotactic radiosurgery (SRS) and stereotactic body radiotherapy (SBRT) where dose gradient is critically evaluated for treatment plans. In this context only small fields were evaluated in this study (≤3 × 3 cm^2^). Figure 6 provides profile data for 6 MV beam for four small field sizes with both detectors compared to Monte Carlo data. For large fields, data is reported by Anders et al.^18^ The differences peaked at the point of inflection for all field size. The differences are minimal (±2%) in beam and towards penumbra. Such large difference is due to steep drop and even 1 mm step can give rise to such large difference which is observed here.

### 3.7 Field output factor

**FIGURE 7:**
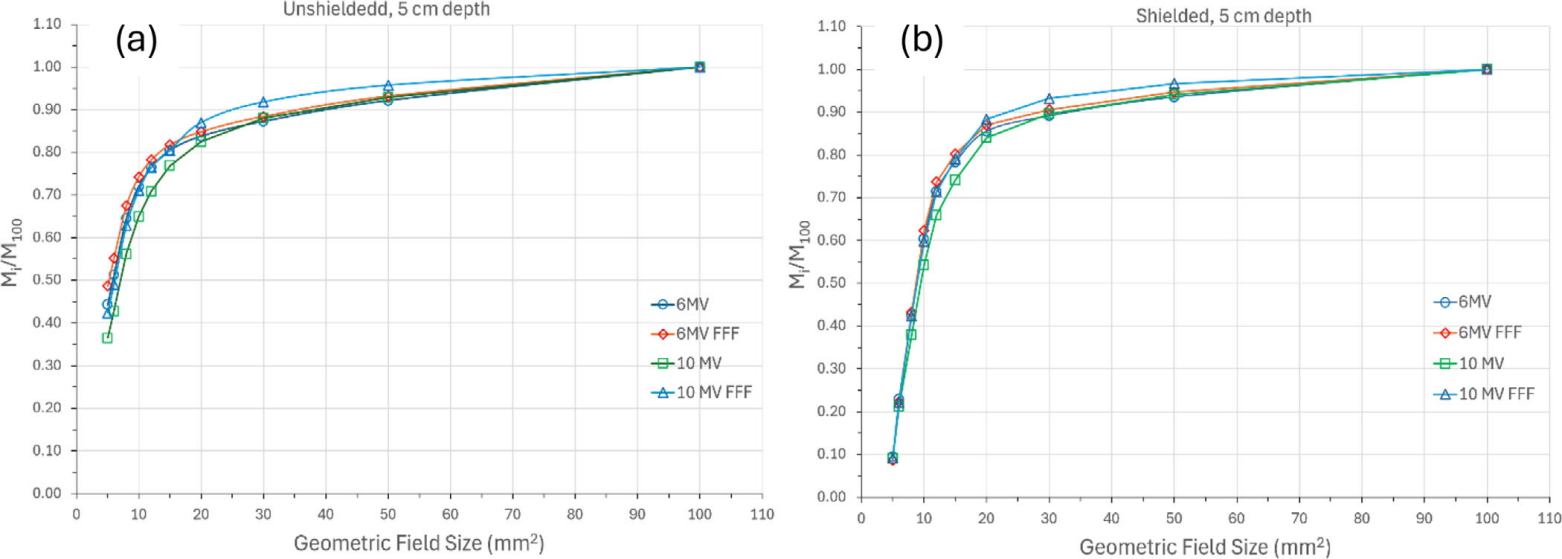
Field output factor in small fields without correction factor (a) unshileded 1048 and (b) shielded 1049 detector.

**FIGURE 8:**
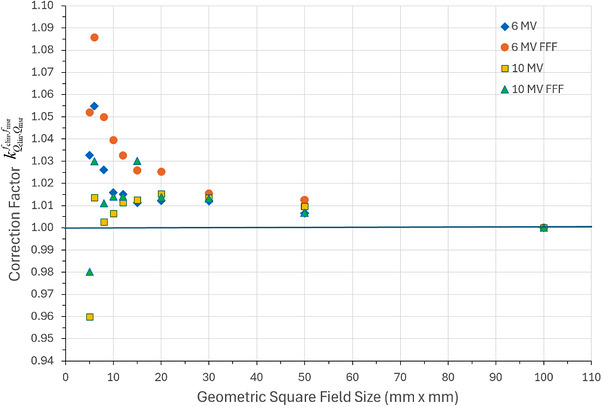
Plot of $k_{Q_{\mathrm{clin}},Q_{\mathrm{msr}}}^{f_{\mathrm{clin}},f_{\mathrm{msr}}}$ versus geometric field size for four beam energies.

As described in the method section, FOF requires $k_{Q_{\mathrm{clin}},Q_{\mathrm{msr}}}^{f_{\mathrm{clin}},f_{\mathrm{msr}}}\mathrm{value}$ which was calculated from Monte Carlo and applied to the SunSILICON detectors very similar to the data shown by Das et al.^27^ The shielded detector model 1049 is meant for large fields, however, we attempted dosimetry in small fields and found that readings fall precipitously for small fields. Figure 7 shows the plot of ratio of readings (M_i_/M_100_) without $k_{Q_{\mathrm{clin}},Q_{\mathrm{msr}}}^{f_{\mathrm{clin}},f_{\mathrm{msr}}}\mathrm{value}.$ For unshielded model 1048, values are very reasonable. The plot of $k_{Q_{\mathrm{clin}},Q_{\mathrm{msr}}}^{f_{\mathrm{clin}},f_{\mathrm{msr}}}$ values derived from MC derived FOF are extracted and are shown in Figure 8 for most photon energies. A term effective field size, *S_clin_* was introduced by Cranmer‐Sargison et al.^33^ to fit the data in small fields that was adapted by TRS‐483.^7^ Since geometric field size is extremely precise for TrueBeam, *S_clin_* is not attempted in this study. Additionally, *S_clin_* is not constant and is variable with individual and detector.^16^, ^26^

The data in Figure 8 shows that SunSILICON detectors have $k_{Q_{\mathrm{clin}},Q_{\mathrm{msr}}}^{f_{\mathrm{clin}},f_{\mathrm{msr}}}$ values >1.0 where a line is drawn. The experimental uncertainty in small field is relatively large (5%‐6%) as indicated by various investigators.^34^, ^35^ However, in this study unshielded detector $k_{Q_{\mathrm{clin}},Q_{\mathrm{msr}}}^{f_{\mathrm{clin}},f_{\mathrm{msr}}}$ value is ±3% for fields > 8 mm^2^ which is within experimental uncertainty. The smallest field 5 mm^2^ is asymmetric (2, 3 mm) and hence data is erratic even though detector was centered based on maximum signal. Table 1 provides data of $k_{Q_{\mathrm{clin}},Q_{\mathrm{msr}}}^{f_{\mathrm{clin}},f_{\mathrm{msr}}}$ excluding 5 mm^2^.

**TABLE 1:** Numerical values of $k_{Q_{\mathrm{clin}},Q_{\mathrm{msr}}}^{f_{\mathrm{clin}},f_{\mathrm{msr}}}$ derived from Monte Carlo for four energies.

Geometric square field size (mm^2^)	6 MV	6 FFF	10 MV	10 FFF
5^a^	1.033^a^	1.052^a^	0.960^a^	0.980^a^
6	1.055	1.086	1.014	1.030
8	1.026	1.050	1.003	1.011
10	1.016	1.040	1.007	1.014
12	1.015	1.033	1.011	1.014
15	1.011	1.026	1.012	1.030
20	1.012	1.025	1.015	1.014
30	1.012	1.015	1.013	1.013
50	1.007	1.013	1.010	1.007
100	1.000	1.000	1.000	1.000

^a^ Erratic data, should be used with caution.

## 4 DISCUSSION

Accurate dosimetry in small photon fields remains one of the most challenging aspects of contemporary radiation therapy, owing to the combined effects of lateral chargedparticle disequilibrium, partial occlusion of the radiation source, detector volume averaging, and detectorspecific perturbations. These challenges are further exacerbated by practical limitations in experimental measurements, including uncertainties in field centering, finite detector positioning accuracy, and stepsize limitations in water phantoms. Although Monte Carlo simulations eliminate many of these experimental constraints, they remain sensitive to voxel size and statistical uncertainty associated with the number of particle histories. Consequently, comprehensive characterization of any new detector is essential before it can be confidently adopted in clinical smallfield dosimetry.

While initial studies have been provided by the group from Sun Nuclear from their perspective,^17^, ^18^ this study provides an indedependent evaluation. In addition, this study provides the small‐field output correction factors that are not availabe for these new SunSILICON detectors.The newly introduced SunSILICON detectors demonstrate highly favorable dosimetric characteristics across a wide range of photon beam energies and field sizes. Both the unshielded (model 1048) and shielded (model 1049) detectors exhibited excellent dose linearity, doserate independence, and minimal temperature dependence within clinically relevant ranges. These fundamental characteristics are critical for routine clinical use, particularly in intensitymodulated radiation therapy (IMRT), volumetricmodulated arc therapy (VMAT), stereotactic radiosurgery (SRS), and stereotactic body radiotherapy (SBRT), where small fields and variable dose rates are routinely employed.

Despite their identical external geometry and sensitive volume, the presence or absence of shielding leads to markedly different dosimetric behavior between the two detectors. The unshielded SunSILICON 1048 detector demonstrated excellent agreement with Monte Carlo simulations for depthdose curves, beam profiles, and field output factors in small fields down to 0.5 × 0.5 cm^2^. The derived smallfield correction factors $k_{Q_{\mathrm{clin}},Q_{\mathrm{msr}}}^{f_{\mathrm{clin}},f_{\mathrm{msr}}}$ for this detector were modest and within ± 3% for field sizes greater than approximately 8 mm^2^, which is comparable to other established smallfield detectors such as plastic scintillators, microSilicon diodes, microDiamond detectors, and radiochromic film. This observation supports the classification of the unshielded SunSILICON detector as a practical, nearcorrectionfree option for smallfield dosimetry when used within its validated range.

In contrast, the shielded SunSILICONP 1049 detector exhibited a pronounced reduction in signal response as the field size decreased below approximately 2 cm. This behavior is consistent with the increased influence of the highZ shielding material under conditions of lateral chargedparticle disequilibrium and limited scatter contribution. While such shielding is effective in mitigating overresponse in medium and large fields—thereby improving water equivalence under electronic equilibrium—it introduces substantial perturbations in small fields. As a result, the required correction factors for the shielded detector become large and highly sensitive to field size, limiting its practical applicability for smallfield dosimetry. These findings are consistent with prior observations for other shielded diode detectors and reinforce current recommendations that shielded diodes be avoided for smallfield measurements.^9^, ^36^

The temperature dependence of both SunSILICON detectors was found to be minimal (<0.5% over a range of 4°C–40°C), which is advantageous for routine clinical measurements performed under standard room and watertank conditions. Compared with other solidstate detectors, including plastic scintillators and diamond detectors, the SunSILICON detectors exhibit similarly stable or superior thermal behavior, eliminating the need for temperature correction factors in most clinical scenarios. This characteristic further simplifies their clinical implementation and reduces potential sources of systematic uncertainty.

Several practical considerations should be noted. Although angular dependence was not explicitly measured in this study, previous investigations have shown it to be within ±1% for clinically relevant orientations. Given that most beam data commissioning and qualityassurance measurements are performed with the detector oriented perpendicular to the beam axis in water phantoms, angular dependence is unlikely to introduce clinically significant error. However, the noncylindrical housing geometry of the SunSILICON detectors poses challenges when used in solid phantoms, as precise machining is required to avoid air gaps that could perturb measured dose. This limitation underscores the importance of careful detector mounting and supports the preferential use of these detectors in waterbased measurement systems.

This study complements and extends previously published manufacturerled characterizations^17^, ^18^ by providing an independent evaluation across multiple photon energies and by explicitly deriving and reporting smallfield correction factors based on Monte Carlo simulations. While largefield depthdose behavior, angular dependence, and radiation damage effects have been addressed in earlier work, the present investigation focuses on clinically relevant smallfield conditions, where detector selection is most critical. Nevertheless, several areas remain open for future study, including detector performance in ultrahigh doserate (FLASH) beams, kilovoltage photon beams, electron beams, proton beams, and brachytherapy applications. Given the high signal output and stability of the SunSILICON detectors, these applications warrant further investigation.

The results of this study emphasize the importance of matching detector design to clinical application. The unshielded SunSILICON 1048 detector offers excellent performance for smallfield photon dosimetry with manageable correction factors, while the shielded SunSILICONP 1049 detector is better suited for conventional medium and largefield applications where electronic equilibrium is maintained. Thoughtful detector selection, informed by an understanding of detectorspecific perturbations, remains essential for achieving accurate and reproducible dosimetry in modern radiation therapy.

## 5 CONCLUSIONS

The dosimetric characteristics of the SunSILICON unshielded (model 1048) and shielded (model 1049) silicon diode detectors were comprehensively evaluated in megavoltage photon beams, with particular emphasis on smallfield dosimetry. Both detectors demonstrated excellent fundamental performance, including high signal output, reproducible response, dose linearity, and minimal dependence on dose rate and temperature over clinically relevant ranges. These features support their suitability for routine relative dosimetry in radiation therapy.

Despite their identical external geometry and sensitive volume, the two detectors exhibit distinctly different clinical applicability owing to the presence or absence of a shielding cover. The unshielded SunSILICON 1048 detector showed favorable performance in small fields, with depthdose, profile, and fieldoutputfactor measurements in good agreement with Monte Carlo simulations. The unshielded detector provides $k_{Q_{\mathrm{clin}},Q_{\mathrm{msr}}}^{f_{\mathrm{clin}},f_{\mathrm{msr}}}$ values within ±3% for field sizes smaller than 0.8 cm^2^for all energies thus FOF can be readily measured and can be treated in the same class of correction free detectors like plastic scintillator detecors, microsilicon, microDiamond and EBT film. The required smallfield correction factors were modest and comparable to those of established smallfield detectors, indicating that model 1048 is well suited for smallfield photon dosimetry across all investigated energies.

In contrast, the shielded SunSILICONP 1049 detector exhibited a pronounced reduction in signal response as field size decreased, leading to large correction factors for small fields. While this behavior limits its applicability in smallfield dosimetry, the shielding effectively reduces overresponse in medium and large fields, making model 1049 appropriate for generalpurpose photonbeam dosimetry where field sizes remain within electronic equilibrium.

Overall, the SunSILICON detector series represents a meaningful advancement in commercial silicondiode technology. The unshielded model provides a reliable option for smallfield dosimetry, while the shielded model is well suited for conventional photonbeam measurements. Careful selection of detector type based on field size and clinical application is essential to ensure accurate dosimetric measurements.

## AUTHOR CONTRIBUTIONS

I.J.D.: Concept, measurement, analysis and writing, M.D.: Measurements, analysis and editing, G.X.D.: Concept, Monte Carlo simulation, data analysis, editing and writing.

## CONFLICT OF INTEREST STATEMENT

The authors declare no conflicts of interest.
